# Spatio-temporal expression of MMP-2, MMP-9 and tissue kallikrein in uteroplacental units of the pregnant guinea-pig (Cavia porcellus)

**DOI:** 10.1186/1477-7827-5-27

**Published:** 2007-07-02

**Authors:** Jenny Corthorn, Sergio Rey, Cecilia Chacón, Gloria Valdés

**Affiliations:** 1 Departamento de Nefrología, Escuela de Medicina, Pontificia Universidad Católica de Chile, Marcoleta 391, 8330024 Santiago, Chile; 2 Centro de Investigaciones Médicas, Escuela de Medicina, Pontificia Universidad Católica de Chile, Santiago, Chile

## Abstract

**Background:**

In humans trophoblast invasion and vascular remodeling are critical to determine the fate of pregnancy. Since guinea-pigs share with women an extensive migration of the trophoblasts through the decidua and uterine arteries, and a haemomonochorial placenta, this species was used to evaluate the spatio-temporal expression of three enzymes that have been associated to trophoblast invasion, MMP-2, MMP-9 and tissue kallikrein (K1).

**Methods:**

Uteroplacental units were collected from early to term pregnancy. MMP-2, MMP-9 and K1 were analysed by immunohistochemistry and Western blot. The activities of MMP-2 and MMP-9 were assessed by gelatin zymography.

**Results:**

Immunoreactive MMP-2, MMP-9 and K1 were detected in the subplacenta, interlobar and labyrinthine placenta, syncytial sprouts and syncytial streamers throughout pregnancy. In late pregnancy, perivascular or intramural trophoblasts expressed the three enzymes. The intensity of the signal in syncytial streamers was increased in mid and late pregnancy for MMP-2, decreased in late pregnancy for MMP-9, and remained stable for K1. Western blots of placental homogenates at days 20, 40 and 60 of pregnancy identified bands with the molecular weights of MMP-2, MMP-9 and K1. MMP-2 expression remained constant throughout gestation. In contrast, MMP-9 and K1 attained their highest expression during midgestation. Placental homogenates of 20, 40 and 60 days yielded bands of gelatinase activity that were compatible with MMP-2 and MMP-9 activities. ProMMP-2 and MMP-9 activities did not vary along pregnancy, while MMP-2 and MMP-9 increased at 40 and 40–60 days  respectively.

**Conclusion:**

The spatio-temporal expression of MMPs and K1 supports a relevant role of these proteins in trophoblast invasion, vascular remodeling and placental angiogenesis, and suggests a functional association between K1 and MMP-9 activation.

## Background

The establishment of pregnancy requires that trophoblasts attach to the uterine epithelium, invade the endometrium, colonize the spiral arteries and acquire an endothelial phenotype, to establish a continuum between the intervillous space and the maternal circulation [1,2]. The disturbance of these tightly spatio-temporally regulated processes causes important obstetrical and neonatal complications, ranging from miscarriages due to impaired attachment, to the extensive invasion of placenta accreta. A shallow invasion, on the other hand, results in a hypoperfused placenta which sheds to the maternal circulation microvillus particles, reactive oxygen species, asymmetric-di-methyl-arginine, cytokines, provoking the preeclampsia syndrome [3,4]. Therefore, the invasive process is critical to determine the fate of pregnancy.

Several studies have provided evidence that invasive trophoblasts secrete matrix metalloproteinases (MMPs), a family of Zn^2+^-dependent endopeptidases that degrade extracellular matrix (ECM) and basement membrane components [5,6]. Two of the most studied members of this family are MMP-2 (gelatinase A, 72 kDa) and MMP-9 (gelatinase B, 92 kDa), which degrade a wide range of substrates, including type IV collagen, the main type of collagen found in basement membranes. MMPs are secreted as proenzymes into the ECM, where they are activated by proteolytic cleavage of their amino terminal domain by membrane-associated MMPs and extracellular proteases. The activity of MMPs is also regulated by binding to different tissue inhibitors of MMPs (TIMPs). MMP-2, MMP-9 and TIMPs are expressed in human, sheep, rat and mice reproductive tissues, and have been implicated in the invasion and development of the placental fetal villous tree; in addition, these proteins participate in the detachment of the placenta and membranes in labor [7-15].

Tissue kallikrein (K1) is a serine protease that cleaves low-molecular weight kininogen to generate kinins (kallidin and bradykinin). Kinins induce vasodilation and increase vascular permeability, either directly or by stimulating the synthesis of nitric oxide (NO) and angiotensin-(1–7) through the type-2 bradykinin receptor (B2R). Moreover, kinins have been shown to enhance mitogenesis and angiogenesis [16]. In humans, K1 and B2R are found in syncytiotrophoblasts, and in invasive and intravascular trophoblasts, along with endothelial NO synthase, angiotensin-(1–7) and angiotensin-converting enzyme type-2 [17,18]. K1 has the capacity to activate MMP-9 in vitro [19-21], and could participate in the degradation of extracellular matrix.

The aim of the present study was to assess the spatio-temporal expression of MMP-2, MMP-9 and K1 in utero-placental units of the pregnant guinea-pig, using immunohistochemistry and Western blot analysis; in addition, the activities of MMP-2 and MMP-9 were evaluated with gelatin zymography. The guinea-pig pregnancy shares several structural similarities with the human gestation, regarding the trophoblastic interstitial and vascular invasion and the haemomonochorial placenta [22-24]. Moreover, the guinea-pig has been postulated as an ideal model to study the role of the tissue kallikrein-kinin system [25,26].

## Methods

Guinea-pigs (Pirbright white ~600 g) were kept under controlled conditions of humidity, temperature and light cycle. Females were examined daily, and when vaginal opening was observed they were caged with fertile males. The day in which sperm was observed in vaginal smears was defined as day one of pregnancy; from then onwards ascorbic acid was added to the drinking water. Litter size varied from 2–5 fetuses.

The experiments were conducted according to the *Guide for the Care and Use of Laboratory Animals *(National Research Council, USA). The animals were deeply anesthetized with a mixture of ketamine (100 mg/kg) and xylazine (4 mg/kg) given intraperitoneally. Thirty dams were sacrificed in early, mid- and late pregnancy. The uterus and feto-placental units were removed and a central slice through the placenta, subplacenta, implantation site and underlying myometrium was fixed as a single block of tissue. In addition, the placenta, endometrium, and mesometrium were separately dissected. The animals were euthanized with an overdose of anesthetic. Tissues were fixed immediately with phosphate-buffered 4% formalin for 24 h or snap frozen in liquid nitrogen and kept at -70°C. The fixed tissues were dehydrated in a graded series of ethanol, xylene and embedded in Paraplast-Plus^® ^(Sigma, St. Louis, MO). Sections (5 μm) were mounted on silanized slides.

### Immunostaining procedure

All immunostaining procedures were performed at room temperature; deparaffinized sections were rehydrated through ethanol, rinsed three times for five minutes in PBS-50 mM Tris-HCl and submitted to heat antigen retrieval using citrate buffer pH 6.0 for K1 and Tris-HCl-EDTA pH 9.0 for MMPs. Endogenous peroxidases were blocked with incubation in 10% H_2_O_2 _for ten minutes. Sections were incubated in a humid chamber for 30 minutes with protein block (Cas-Block^®^, Zymed, San Francisco, CA) followed by incubation for 18 h at 4°C with the primary antibodies: mouse monoclonal anti-MMP-2 (clone 75-7F7, 2 μg/ml), anti-MMP-9 (clone 56-2A4, 2 μg/ml) from Oncogene Research, San Diego, CA and rabbit polyclonal antiserum against purified rat urinary K1 (1:2000) [27]. These antibodies recognize both active and latent forms. Sections were immunostained using a biotin-streptavidin-peroxidase system (LSAB+^®^, DakoCytomation, Carpinteria, CA). Finally, the samples were treated for 15 minutes with 0.1% (w/v) 3-3'-diaminobenzidine in buffer containing 0.05% H_2_O_2_. The slides were counterstained with Harris hematoxylin (Sigma, St. Louis, MO).

Cytotrophoblasts were identified by staining with an anti-pancytokeratin mouse monoclonal antibody (1:100, P2871, Sigma, St. Louis, MO). Smooth muscle and endothelial cells were characterized with antibodies against α-smooth muscle actin (1A4, 1:1500), muscle actin (clone HHF35, 1:1000) and von Willebrand factor (A0082, 1:400) obtained from DakoCytomation (Carpinteria, CA). The specificity of the staining was determined by incubation of sections in the absence of the first antibody.

### Immunohistochemical image analysis

The immunostained sections of the subplacenta and decidua were photographed with a Nikon CoolPix 4500 (Nikon Inc., Tokyo, Japan) camera coupled to a Zeiss AxioImager AX.10 microscope (Carl Zeiss, CA) using a 20× objective with a 0.32 μm/pixel resolution. The luminance of the incident light was calibrated for each section in order to assign pixel values from 0 to 255 (no light to full light transmission). A total of one hundred and forty five subplacental and decidual fields were photographed from sections obtained from dams in each of the following periods: early (20 days), mid (40 days) and late (60 days) pregnancy. A total of 12–16 fields were analyzed for MMP-2, MMP-9 and K1 immunohistochemistry at each gestation period. The images were loaded into the ImageJ v.1.34 software (National Institutes of Health, Bethesda, MD) for analysis. A total of 243 syncytial streamers were manually delineated, extracted from the surrounding tissue to a separate image file and saved in tiff format. MMP-2, MMP-9 and K1 immunoreactive signals were extracted from the images with a color deconvolution algorithm [28], integrated in the ImageJ software. After conversion of pixel luminosity values to an optical density scale, the integrated optical density was measured in the previously extracted positive staining images and normalized by the positive area in each microphotograph. The signal intensity (I) was calculated as I = 10·∑OD/A in dB/μm^2^, being ∑OD the integrated optical density, and A the area of positive staining (μm^2^).

### Protein extraction

Total proteins from placenta from early, mid and late pregnancy were extracted using 5 ml/g of 20 mM Tris-HCl buffer containing 10 mM EDTA, 2 mM phenylmethylsulfonylfluoride, 5 μM leupeptin, 50 μg/ml soybean trypsin inhibitor, 0.05% Brij-35 and 0.02% NaN_3 _at pH 7.4. Tissues were homogenized with a Tekmar Tissumizer (Cincinnati, OH) for one minute on ice. Crude homogenates were centrifuged at 4000 rpm for 20 minutes, then 0.2% SDS was added to the supernatant, kept for 1 hour at 4°C, centrifuged at 14000 rpm for 20 min at 4°C and finally stored at -70°C. Protein content was determined according to the Lowry method [29].

### Western blot analysis

Equal amounts of protein (100 μg/lane) were separated using 10% SDS-PAGE under reducing conditions and transferred to nitrocellulose membranes (Biorad, Hercules, CA), blocked with 5% nonfat dry milk in PBS-0.1% Tween-20 buffer (PBS-T) and incubated overnight at 4°C with the same primary antibodies used in immunohistochemistry: anti-MMP-2 (1 μg/ml), anti-MMP-9 (1 μg/ml) or anti-K1 (1:8000) diluted in blocking buffer.

Standards were purified human recombinant proMMP-2, proMMP-9 (20 ng, Calbiochem, La Jolla, CA), and purified rat urinary kallikrein (5 ng) [27]. The membranes were washed six times for five minutes in PBS-T buffer, incubated with HRP-conjugated anti-mouse or anti-rabbit secondary antibodies (both 1:3000, Biorad, Hercules, CA) for one hour at room temperature and developed with chemiluminescence reagent (NEL-103, Western Lighting, Perkin-Elmer, MA) [30]. Membranes were exposed to CL-xPosure film (Pierce, Rockford, IL). Equal protein loading was confirmed with Ponceau-S red staining (Sigma, St. Louis, MO). Images were scanned at 16-bit/600dpi resolution with an Epson Perfection 3490 scanner (Epson Corporation, CA), saved as tiff files and calibrated to an optical density scale. The integrated optical density of bands was quantitated using the ImageJ v.1.34 software and expressed as fold- values of the average optical density at day 20 ± SE for K1. For MMP-2 and MMP-9, the optical densities were expressed as the ratio of active/total enzyme (total equals the sum of the optical densities of the bands).

### Gelatin zymography

Gelatinase activity was detected by zymography using methods described previously with some modifications [31]. Placental proteins (100 μg/lane) were resolved under non reducing conditions in an 8% polyacrylamide gel containing 0.5 mg/ml gelatin (porcine skin, 300-bloom, Sigma, St. Louis, MO). After electrophoresis, the gels were washed twice at room temperature for 30 minutes in 2.5% Triton X-100, subsequently washed in buffer containing 50 mM Tris-HCl, 150 mM NaCl, 5 mM CaCl_2_, 1 μM ZnCl_2_, 0.05% Brij-35, 0.02% NaN_3 _at pH 7.5 and incubated in this buffer at 37°C for 24 h. Thereafter, the gels were stained with 0.2% (w/v) Coomassie brilliant blue R-250 (Sigma, St. Louis, MO) for one hour, lightly destained in methanol:acetic acid:water (3:1:6) and finally stored in 5% acetic acid. Identification of each gelatinase band was done in accordance to their molecular weight using purified human recombinant proenzymes MMP-2 and MMP-9 as standards (0.25 ng). Gels were scanned in transmissive mode at 16-bit color/600dpi (Epson Perfection 3490, Epson, CA) and stored in tiff format. Images were processed extracting the blue channel signal, converted to black and white and inverted for quantitation of the integrated optical density of gelatinolytic activities using the ImageJ v.1.34 software. Data was expressed as fold-values of the average optical density at day 20.

### Statistical analysis

Overall differences between the three studied periods of pregnancy were assessed with the Kruskal-Wallis test with *posthoc *unpaired comparisons using the Dwass-Steel-Chritchlow-Fligner (DSCF) test. All analyses were performed with the StatsDirect v.2.6.1 software (StatsDirect Ltd., Cheshire, UK). Statistical significance was set at *P *< 0.05. Results are expressed as means ± SE.

## Results

Spatio-temporal expression of MMP-2, MMP-9 and K1 in uteroplacental units

### Subplacenta

**Figure 1:**
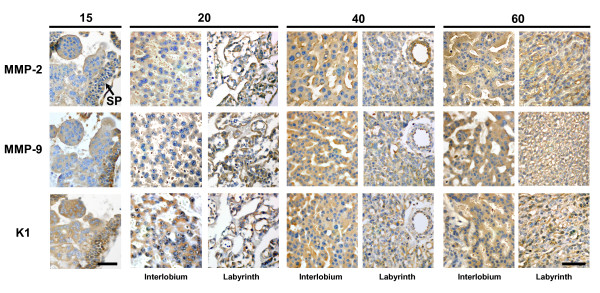
In subsequent sections of a uteroplacental unit obtained in day 15 of pregnancy, the multilayered subplacenta (SP), and the initial sprouts that give origin to the placenta, expressed K1, MMP-2 and MMP-9. Syncytiotrophoblasts composing the interlobium and the labyrinth expressed K1, MMP-2 and MMP-9 in sections obtained in days 20, 40 and 60 of pregnancy. Bar = 50 μm.

The folded multilaminar layer of cytotrophoblasts that constitutes the subplacenta appeared to be the main, if not the exclusive, source of trophoblasts. Clusters of syncytiotrophoblast, which constituted the earliest expression of the placenta, were observed at day 15 of gestation towards the uterine lumen of the subplacenta. From its base also rose syncytial sprouts, which penetrated the endometrium. At this stage, the subplacental cytotrophoblasts expressed MMP-2, MMP-9 and K1 (Fig. 1). The positive subplacental immunoreactivity was observed throughout pregnancy. The groups of multinucleated giant cells found in the junctional zone close to the decidua also showed staining for these enzymes (not shown).

### Placenta

As early as day 15, trabeculae and syncytiotrophoblast plates originated from the subplacenta, and expressed immunoreactivity for MMP-2, MMP-9 and K1. This granular staining persisted in the thick bands of syncytiotrophoblast of the interlobium and in the thin trabeculae composed by syncytium and endothelial cells of the labyrinth, from their development to term (Fig. 1).

**Figure 2:**
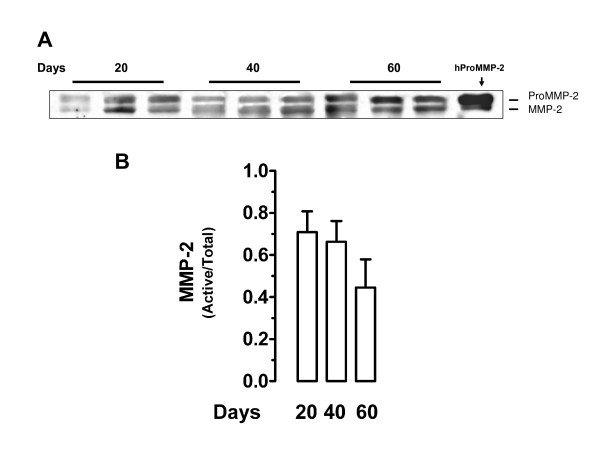
Western blot analysis of guinea-pig placental homogenates for MMP-2. **A**, Representative blot at days 20, 40 and 60 of pregnancy. Purified human proMMP-2 (hProMMP-2) yielded two bands with approximate molecular weights of 72 and 62 kDa. **B**, Mean ratio of active/total MMP-2 in three placental homogenates for each period showed no significant differences. DSCF *posthoc *comparisons after Kruskal-Wallis test.

**Figure 3:**
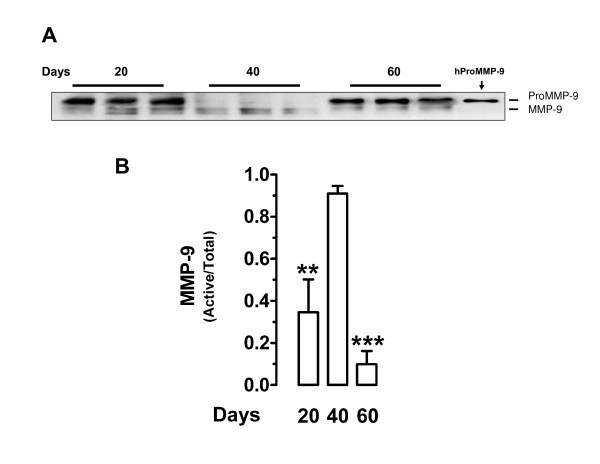
Western blot analysis of guinea-pig placental homogenates for MMP-9. **A**, Representative blot at days 20, 40 and 60 of pregnancy. Purified human proMMP-9 (hProMMP-9) yielded two bands with approximate molecular weights of 92 and 68 kDa. **B**, Mean ratio of active/total MMP-9 in five placental homogenates for each period. **P < 0.01 for 20 *vs*. 40 days; ***P < 0.001 for 40 *vs*. 60 days. DSCF *posthoc *comparisons after Kruskal-Wallis test.

**Figure 4:**
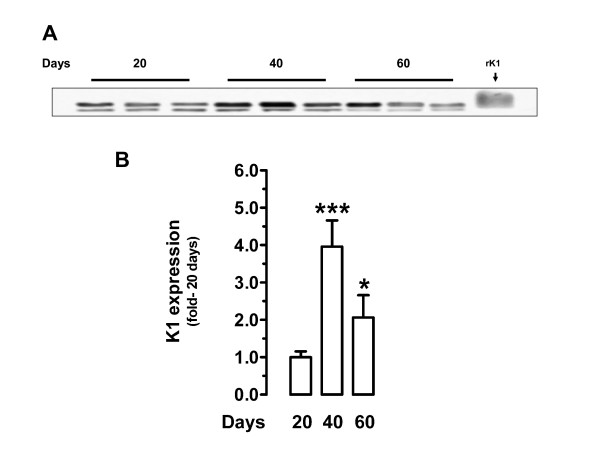
Western blot analysis of guinea-pig placental homogenates for tissue kallikrein (K1). **A**, Representative blot at days 20, 40 and 60 of pregnancy. Purified rat K1 (rK1) was detected as a broad band with an approximate molecular weight of 31 kDa. **B**, Densitometric analysis of K1 expression in five placental homogenates for each period. Data is expressed with respect to the average densitometry at day 20. *, P < 0.05 for 60 *vs*. 40 days; ***, P < 0.001 for 40 *vs*. 20 days. DSCF *posthoc *comparisons after Kruskal-Wallis test.

Western blot analysis of placental homogenates at days 20, 40 and 60 of pregnancy identified bands corresponding to the molecular weights of MMP-2, MMP-9 and K1. The bands corresponding to pro and active MMP-2 did not show a significant change of expression as pregnancy progressed (Fig. 2A); as expected, the ratio of active/total MMP-2 remained constant along pregnancy (Fig. 2B). ProMMP-9 expression decreased at day 40 (*P *< 0.05) while active MMP-9 attained its lowest expression at day 60 (*P *< 0.05; Fig. 3A). The ratio of active/total MMP-9 increased significantly at mid- pregnancy (*P *< 0.01; Fig. 3B), in parallel to the highest K1 expression level (*P *< 0.05; Fig. 4A,B).

**Figure 5:**
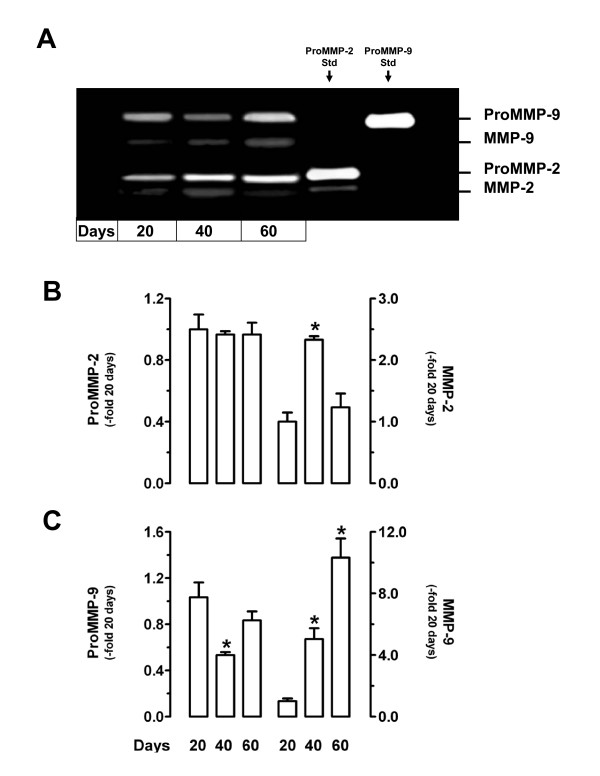
Gelatin zymography of placental homogenates. **A**, Representative zymogram at days 20, 40 and 60 of pregnancy. Purified human proMMP-2 (hproMMP-2) and proMMP-9 (hproMMP-9) were used as standards. **B-C**, Densitometric analysis of zymograms from three placental homogenates at days 20, 40 and 60 for Pro/MMP-2 (B) and Pro/MMP-9 (C). Data is expressed with respect to the average densitometry at day 20. *, P < 0.05 for 40 *vs*. 20 and 60 days in (B); *, P < 0.05 for 40 and 60 *vs*. 20 days in (C). DSCF *posthoc *comparisons after Kruskal-Wallis test.

Gelatinase activity was detected in homogenates of placentae at 20, 40 and 60 days of pregnancy, with two bands of 72 and 92 kDa, thus matching the predicted size of the latent forms of MMP-2 and MMP-9. Another two bands with weights ~10 kDa lower than the latent forms were detected, probably corresponding to the active forms of MMP-2 and MMP-9. ProMMP-2 and ProMMP-9 gelatinase activities did not show variations along pregnancy, while active MMP-2 increased at 40 days (*P *< 0.05), and active MMP-9 increased at 40 and 60 days (*P *< 0.05) compared to day 20 of pregnancy (Fig. 5A,B,C).

### Endometrium

As early as day 15 of gestation, cytokeratin positive cells were observed in the capillary walls, and invasive trophoblasts coalesced into syncytial streamers, or finger-like columns migrating into the endometrium. From day 20 onwards, plugs were observed in some arterial lumina (not shown).

**Figure 6:**
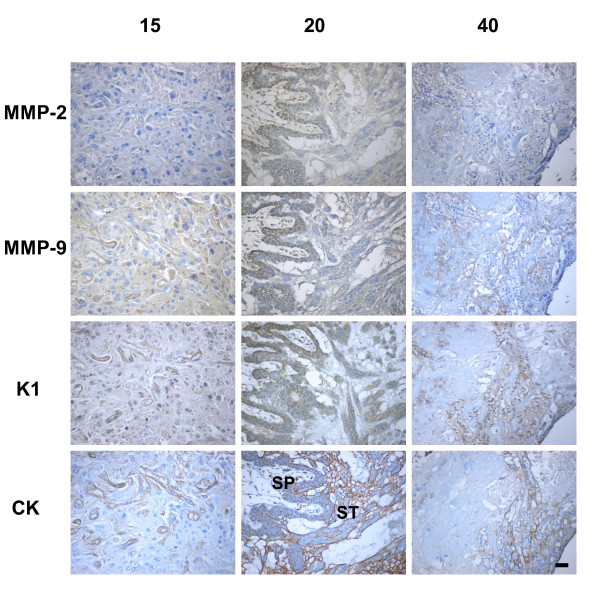
Trophoblasts characterized by cytokeratin (CK) in the subplacenta (SP) and in the decidual syncytial streamers (ST) expressed MMP-2, MMP-9 and K1 in sequential sections of days 15, 20 and 40 of pregnancy. Bar = 50 μm

MMP-2, MMP-9 and K1 immunoreactivity in streamers showed a granular staining (Fig. 6). The area occupied by streamers, quantified by digital analysis, was increased in midpregnancy, as compared to the early and late stages (1,163 ± 62 versus 944 ± 91 and 644 ± 39 μm^2^, *P *< 0.01). In addition, the signal intensity increased in mid and late pregnancy for MMP-2 (4.1 ± 0.2 and 4.0 ± 0.1 versus 3.1 ± 0.2 dB/μm^2 ^in early pregnancy, *P *< 0.05), decreased in late pregnancy for MMP-9 (3.0 ± 0.1 versus 3.6 ± 0.2 and 3.8 ± 0.2 dB/μm^2 ^in early and mid- pregnancy, *P *< 0.05), and remained stable for K1 (pooled average 4.0 ± 0.1 dB/μm^2^).

### Spiral and mesometrial arteries

**Figure 7:**
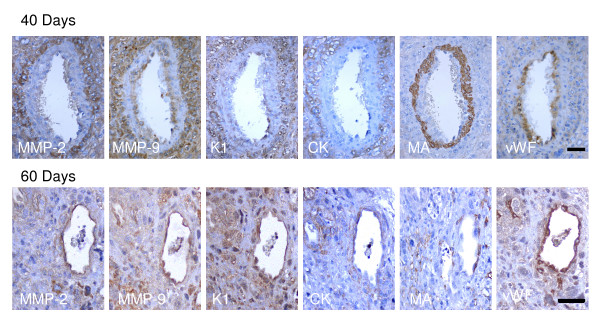
Spiral arteries observed in sequential sections of myometrium obtained in days 40 and 60 of pregnancy, expressed MMP-2, MMP-9 and K1 in trophoblasts surrounding the vessel, characterized by cytokeratin (CK), and in the swollen endothelial cells, characterized by von Willebrand Factor (vWF). The remaining vascular smooth muscle in day 40 was positive for muscle actin (MA), and has been replaced in day 60. Bar = 50 μm

Spiral arteries in mid- pregnancy were surrounded by cytotrophoblasts, which had partly disrupted the vascular smooth muscle layer, and expressed MMP-2, MMP-9 and K1. These enzymes were also observed in swollen endothelial cells. In late pregnancy cytotrophoblasts replaced the smooth muscle layer and attained the luminal border; these cells presented a diffuse staining for MMP-2 and a granular staining for MMP-9 and K1. Endothelial cells, identified by vWF were swollen, and expressed the three enzymes (Fig. 7).

**Figure 8:**
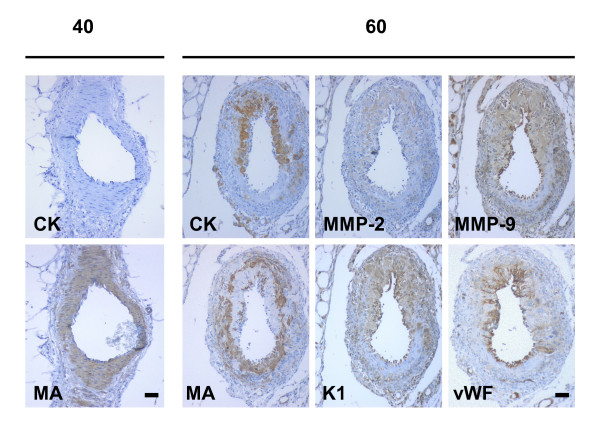
Mesometrial arteries obtained in day 40 of pregnancy showed in sequential sections an intact smooth muscle layer characterized by muscle actin (MA), and no cytokeratin positive cells. In day 60, intramural trophoblasts, characterized by cytokeratin (CK), expressed MMP-2, MMP-9 and K1, while endothelial cells, identified by von Willebrand Factor (vWF), were swollen, and expressed MMP-9 and K1. The remaining vascular smooth muscle, positive for muscle actin (MA), was disrupted. Bar = 50 μm

Mesometrial arteries in early and mid pregnancy had a thick multilayer zone of smooth muscle cells and no trophoblasts were observed in their periphery or within the muscle layer. In late pregnancy trophoblasts replaced part of the muscle layer, and presented a positive signal for MMP-2, MMP-9 and K1. Endothelial cells, identified by vWF, were swollen and expressed MMP-9 and K1 (Fig. 8).

**Figure 9:**
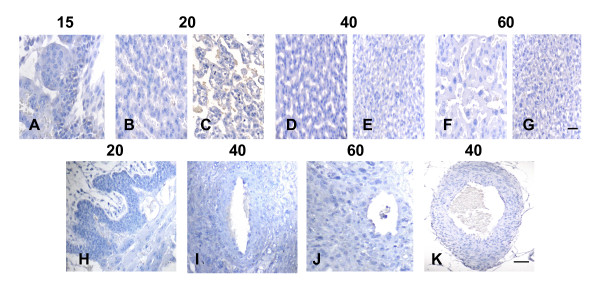
Control sections at different days of pregnancy, incubated in absence of the first antibody for subplacenta (A), interlobium and labyrinth (B-G), subplacenta and syncitial streamers (H), spiral arteries (I,J) and mesometrial artery (K). Bar = 50 μm

Control sections incubated in absence of the first antibody yielded no staining in different structures and stages of pregnancy (Fig. 9).

## Discussion

The present study represents the first demonstration of the expression of MMPs and K1, as well as their coincident localization in different cell types of the feto-maternal interface of the guinea-pig. The spatio-temporal localization of these enzymes in critical sites and periods of the placentation process, as well as the modulation in the activity of MMPs, suggest a functional role in relation to trophoblast invasion and placental angiogenesis.

The immunohistochemical expression of MMP-2 and MMP-9 in placental syncytiotrophoblasts, subplacenta and invading trophoblasts in the guinea-pig is similar to that reported in the human feto-maternal interface in the syncytium, anchoring columns and invasive trophoblasts respectively [11-15], cell types that perform similar functions. This statement derives from the fact that the subplacental trophoblasts contact the uterine wall and are the main – if not exclusive – source of invading trophoblasts, thus being comparable to the anchoring villi; the syncytial streamers of the guinea-pig, though fused, represent the invasive trophoblasts, and the peri and intraarterial trophoblasts are shared by both species.

Since the antibodies used for immunohistochemistry in the present study do not differentiate active from inactive MMPs, the addition of Western Blot and zymography of placental homogenates showed a modulation of the enzymes. A more exact analysis of the variations of MMP activity in the different structures of the utero-placental unit needs to be pursued using *in situ *gelatin zymography.

In syncytial streamers, the immunoreactive expression of MMPs, semiquantified by digital analysis, increased for MMP-2 in mid- and late pregnancy, while for MMP-9 decreased in late pregnancy. The variations of the immunoreactivity in streamers, support a temporal regulation. In humans, data derived from placental sections and from trophoblasts in cultures [11,32] also show temporal variations of MMP-2 and MMP-9.

The immunoreactive expression of K1 in placenta, subplacenta, invasive and intraarterial cytotrophoblasts of the guinea-pig is also similar to that found in equivalent cell types and structures of the human utero-placental unit [17]. In placenta, K1 was increased in mid- and late pregnancy. The intensity of the immunoreactive signal for K1 in syncytial streamers showed no variation along the different stages of pregnancy. It is important to relate the expression of K1 to that of the B2R, the mediator of the classical vasodilator effect of the tissue kallikrein-kinin system. This receptor has been found by our group in cytotrophoblasts of basal syncytial sprouts, in syncytial streamers, in perivascular trophoblasts, and in placental syncytium and endothelium [33]. The localization of the enzyme and receptor of its main peptide support our hypothesis that kinins favor angiogenesis and vasodilation.

Several authors have found that K1 activates MMP-9 *in vitro *[19-21], therefore the coincident localization of MMP-9 and K1, as well as the higher ratio of active/total MMP-9 parallel to the highest expression of K1, favors a functional association that could regulate MMP activation in trophoblasts, and modify their invading capacity.

Trophoblast invasion shares many processes and molecules with the proliferation and dispersion of cancer cells [34]. MMPs have been extensively described in tumoral cells, and are thought to be key facilitators of cell spreading. K1 has also been recognized in several types of cancers, and has been related to the capability of kinins to enhance mitosis, angiogenesis and vascular permeability [16,35]. As stated above, and considering the increased K1 immunolabelling in extravillous trophoblast in placenta accrete [17], we hypothesize that K1 may also contribute to trophoblast invasion.

It is important to consider that MMPs are absolutely necessary for vessel development. In tumors, MMP-2 and MMP-9 play a critical role in the "angiogenic switch" [36]. The increased expression of the active form of MMP-9 in day 40 of gestation, in a stage of rapid placental growth [22], could be associated with enhanced vessel development. As to the kallikrein-kinin system, angiogenic properties have been attributed to bradykinin [37,38].

Having analyzed a possible role for MMPs and K1 regarding the invasion of the uterine stroma and placental angiogenesis, the extensive penetration of trophoblasts through the wall of spiral and mesometrial arteries observed in the guinea-pig [39] deserves to be commented in the same light. The increasing number of perivascular and intramural cytotrophoblasts in mid and late pregnancy in spiral arteries, and the intramural trophoblasts in late pregnancy in mesometrial arteries, expressed the triad of MMP-2, MMP-9 and K1. As endovascular trophoblast incorporation very likely depends on the same mechanisms of interstitial stromal invasion, the activation of MMP-9 by K1 may enhance the disruption of the subendothelial basement membranes and other matrix components of the vascular wall. We would like to emphasize that in spite of occasional intravascular plugs, the main route of vascular invasion in the guinea-pig is the interstitium.

The interspecies conservation of MMPs and K1 immunolocalization, the coincident localization of MMPs and K1, and the modulation of these enzymes along pregnancy, supports a relevant and interrelated role of these proteins in placental development. Though it is known that inhibition of MMPs impairs trophoblast invasion *in vitro *[40], there are no current studies of the effect of kallikrein and/or B2R inhibitors on trophoblast invasion. We believe that the interrelated roles of MMPs and K1 supported by the present findings should motivate further studies.

## Competing interests

The author(s) declare that they have no competing interests.

## Authors' contributions

The study design, the histological analysis, the interpretation of the data and the drafting of the manuscript were done by JC and GV, the animals were sacrificed by JC and CC; the immunocytochemistry was performed by JC and CC, western blotting and zymographies were done by JC, image and densitometrical analysis were done by SR. All authors read and approved the final manuscript.
